# Inherited DNA Repair Gene Mutations in Men with Lethal Prostate Cancer

**DOI:** 10.3390/genes11030314

**Published:** 2020-03-14

**Authors:** Tommi Rantapero, Tiina Wahlfors, Anna Kähler, Christina Hultman, Johan Lindberg, Teuvo L. J. Tammela, Matti Nykter, Johanna Schleutker, Fredrik Wiklund

**Affiliations:** 1 Faculty of Medicine and Health Technology, Prostate Cancer Research Center, Tampere University, 33100 Tampere, Finland; tommi.rantapero@tuni.fi (T.R.); tiina.wahlfors@veripalvelu.fi (T.W.); teuvo.tammela@pshp.fi (T.L.J.T.); matti.nykter@tuni.fi (M.N.); 2 Department of Medical Epidemiology and Biostatistics, Karolinska Institutet, 17177 Stockholm, Sweden; anna.kahler@ki.se (A.K.); christina.hultman@ki.se (C.H.); johan.lindberg@ki.se (J.L.); 3 Institute of Biomedicine, University of Turku, 20014 Turku, Finland; johanna.schleutker@utu.fi; 4 Department of Medical Genetics, Genomics, Laboratory Division, Turku University Hospital, 20521 Turku, Finland

**Keywords:** prostate cancer, DNA repair genes, lethal cancer

## Abstract

Germline variants in DNA repair genes are associated with aggressive prostate cancer (PrCa). The aim of this study was to characterize germline variants in DNA repair genes associated with lethal PrCa in Finnish and Swedish populations. Whole-exome sequencing was performed for 122 lethal and 60 unselected PrCa cases. Among the lethal cases, a total of 16 potentially damaging protein-truncating variants in DNA repair genes were identified in 15 men (12.3%). Mutations were found in six genes with *CHEK2* (4.1%) and *ATM* (3.3%) being most frequently mutated. Overall, the carrier rate of truncating variants in DNA repair genes among men with lethal PrCa significantly exceeded the carrier rate of 0% in 60 unselected PrCa cases (*p* = 0.030), and the prevalence of 1.6% (*p* < 0.001) and 5.4% (*p* = 0.040) in Swedish and Finnish population controls from the Exome Aggregation Consortium. No significant difference in carrier rate of potentially damaging nonsynonymous single nucleotide variants between lethal and unselected PrCa cases was observed (*p* = 0.123). We confirm that DNA repair genes are strongly associated with lethal PrCa in Sweden and Finland and highlight the importance of population-specific assessment of variants contributing to PrCa aggressiveness.

## 1. Introduction

Prostate cancer (PrCa), the most common male cancer worldwide, has a wide spectrum of clinical behavior that ranges from decades of indolence to rapid metastatic progression and lethality [1]. PrCa is also among the most heritable human cancers, with 57% of the interindividual variation in risk attributed to genetic factors [2]. Genome-wide association studies (GWAS) have thus far confirmed ~170 susceptibility loci that account for over 30% of the familial relative risk [3]. However, the risk variants identified using case-control designs show little or no ability to discriminate between indolent and fatal forms of this disease [4]. Therefore, studies contrasting patients with more and less aggressive disease and those exploring associations with disease progression and prognosis should be more effective at detecting genetic risk factors for aggressive PrCa with prognostic potential.

Inherited and acquired defects in DNA repair genes are a common hallmark of cancer and, to date, numerous inherited DNA repair gene mutations that increase cancer risk has been identified [5]. In particular, mutations in *BRCA1* and *BRCA2* genes, both associated with several DNA repair pathways, confer a strikingly increased risk of breast and ovarian cancer [6]. In addition, it is now recognized that the downregulation of DNA repair response is necessary for tumor progression into a more aggressive phenotype [5]. Accumulating evidence suggests that pathogenic germline variants in known cancer-predisposing genes such as *BRCA2* can increase the risk of developing PrCa, especially the more aggressive form of the disease [7]. Likewise, several other genes that were initially implicated as high-risk genes in cancers other than PrCa, such as *CHEK2* and *BRIP1,* have subsequently been shown to increase the risk of PrCa as well [8,9,10]. Recent studies have reported a high carrier rate of inherited DNA repair gene mutations among men with metastatic PrCa (11.8%), significantly exceeding the prevalence (4.6%) among men with localized PrCa [11].

In this study, we evaluated germline variants of DNA repair genes in men who died of PrCa. The aim of our study was to identify and investigate the frequency of pathogenic germline variants in men with the lethal form of the disease.

## 2. Materials and Methods 

### 2.1. Study Subjects

Genomic DNA from a total of 122 lethal PrCa patients was collected from an ongoing collection of Finnish PrCa patients (TAMPERE, *n* = 47) and the Swedish Cancer of Prostate in Sweden (CAPS, *n* = 75) study. To create an extremely aggressive phenotype, the inclusion criterion for lethal PrCa cases was that the patient should have died due to PrCa before the age of 65. All of the Finnish patients were recruited in the Pirkanmaa Hospital District as part of a hereditary PrCa family collection or through collection of sporadic cases treated at the regional hospital [12]. The Swedish CAPS study is a population-based case-control study that enrolled participants between 2001 and 2003 [13]. An additional 70 PrCa patients from the TAMPERE population, not selected for disease aggressiveness or young age at death (hereby denoted unselected cases), with whole-exome sequencing data available were also included to contrast against the lethal cases. Clinical information, such as clinical stage, pathologic grade, nodal or distant metastases, and diagnostic serum levels of PSA and vital status, including cause of death, was obtained through medical records and national cancer registries. All samples were collected with written and signed informed consent. The project was approved by the research ethics committee at Pirkanmaa Hospital District (R03203), the Finnish National Supervisory Authority of Welfare and Health (5569/32/300/05) and by the ethics committees at the Karolinska Institutet (04-449/4 and 06-381/32).

### 2.2. Sample Preparation, Sequencing and Genotyping

Genomic DNA was extracted from whole blood by standard methods. For the 122 lethal cases, exome capture was performed using Agilent SureSelect Human All Exon 50 M kit (Agilent Technologies, Inc., Santa Clara, CA, USA) according to standard protocol and sequenced at the Science for Life laboratory (Stockholm, Sweden). Of the 70 unselected cases 25 samples were sequenced by BGI Tech Solutions (Hong Kong, China) with exome capture performed by the SureSelect Human All Exon 50 M kit while the remaining 45 unselected cases were sequenced at Mayo Clinic, Rochester, MN, USA with exome capture performed using Agilent SureSelect Human All Exon 50Mb or V4+UTR kits. At each site samples were sequenced using the Illumina Hiseq (Illumina, Inc, San Diego, CA, USA). 

### 2.3. Sample Quality Control and Variant Calling

The reads were aligned against the hg19 genome build retrieved from UCSC using BWA [14]. BEDtools [15] was used to calculate the genome-wide coverage for each sample where samples with less than 30% of bases covered by at least 20 reads were excluded. The PCR duplicates were marked using PICARD [16], and the base score recalibration was performed using GATK [17]. Subsequently, GATK was used to call the variants and genotypes following the GATK best practices protocol for germline exome-sequencing data [18,19]. The candidate false-positive variants were initially filtered using the variant quality score recalibration procedure using the tranche threshold 99.0. Furthermore, variants having an allele fraction of less than 0.3 or a coverage of less than 12 were filtered out. Finally, variants with a readPosRankSum less than or equal to −1.7 were discarded. The variants were annotated using ANNOVAR [20].

### 2.4. Variant Prioritization

Variants found in 175 DNA repair genes [21,22,23] were selected for further analysis. To prioritize variants for validation, we utilized a similar approach to that introduced by Mijuskovic and coworkers [7]. The intergenic and common (minor allele frequency > 0.01) variants were filtered out. The remaining rare variants were classified into two categories: potentially damaging and neutral. The potentially damaging variants were further classified into two categories (Tier 1 and Tier 2) based on their impact. The classification was performed utilizing a database of reported associations of variants to clinical phenotypes (ClinVar) provided by ANNOVAR and two tools for pathogenicity prediction, CADD [24] and REVEL [25], of which the latter is specifically designed for discovery of rare deleterious variants. Moreover, the known protein domains from the UniProt [26] database were utilized to assess the pathogenicity of protein truncating variants.

**Figure 1:**
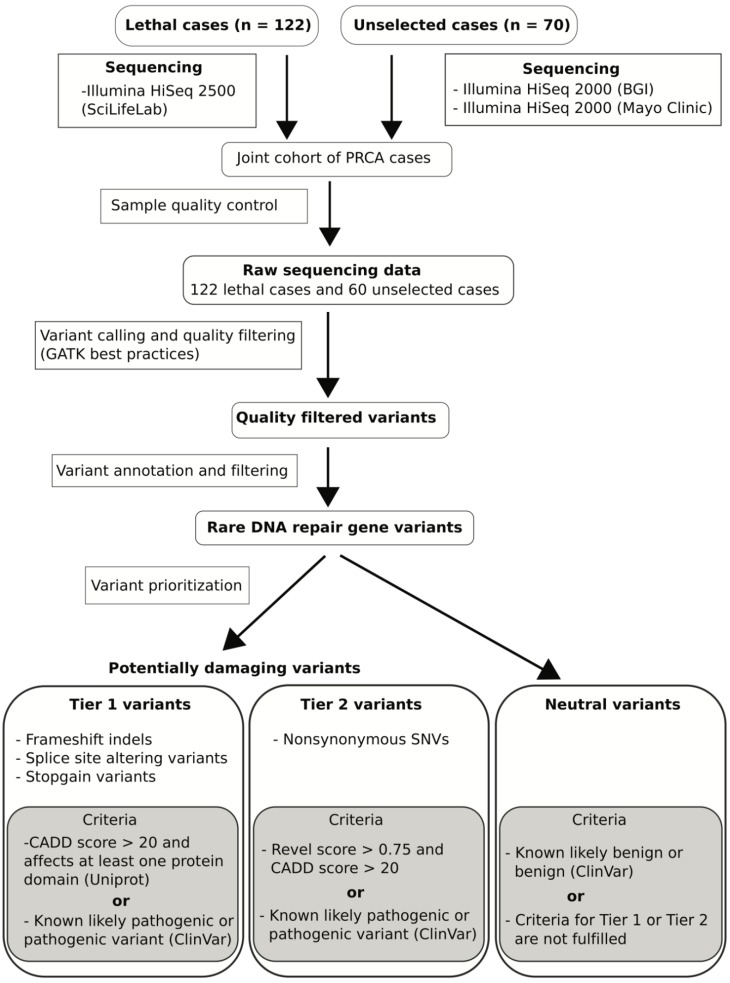
Flow chart describing processing of whole exome sequencing, quality control, variant calling and annotation, and variant prioritizing. PRCA: prostate cancer; ClinVar: database of reported associations of variants to clinical phenotypes; CADD: combined annotation dependent depletion; Revel: rare exome variant ensemble learner.

Those variants that are reported as likely benign or benign in ClinVar were classified as neutral. Protein truncating variants (stopgain, frameshift indels or splicing site altering variants) were classified as Tier 1 variants if they had a CADD phred score ≥ 20. Furthermore, the variants were required to be reported to be pathogenic or likely pathogenic by the ClinVar database or alternatively known to affect a protein domain reported in Uniprot (e.g., occurring before or within a protein domain). All nonsynonymous single nucleotide variants (missense variants) reported to be pathogenic or likely pathogenic by ClinVar or had a CADD phred score ≥ 20 and REVEL score ≥ 0.75 were classified as Tier 2 variants. The same prioritization criteria were applied to both case cohorts. The full workflow including details of the sequencing data analysis is illustrated in Figure 1.

### 2.5. Population Frequencies

To explore the expected population allele frequencies of pathogenic variants in the discovered DNA repair genes, we extracted data from two subsets of the Exome Aggregation Consortium (ExAC) browser [27], one set comprising 6192 Swedish population controls and one set comprising 3307 Finnish individuals unselected for cancer history. Full details of the data processing, variant calling and resources have been described previously [27]. Variant prioritization among these population controls was performed by the same filtering algorithm as described above for the PrCa cases.

### 2.6. Statistical Analysis

Baseline characteristics were described using the median (interquartile range [IQR]) for continuous variables and absolute and relative frequencies for categorical variables. The frequency of potentially damaging DNA repair gene mutation carriers among the lethal PrCa patients was compared to the frequency in unselected PrCa patients and the two control populations with the use of a two-sided Fisher’s exact test. For the control populations, the frequency of mutation carriers in a specific gene was calculated on the basis of the total number of persons for whom sequence coverage was adequate for the given allele, under the assumption that each individual carried at most one deleterious mutation in the explored gene. This assumption may have introduced a slight overestimation in the carrier frequency in the control populations. In all analyses, Tier 1 and Tier 2 mutations were assessed separately. No adjustment was made for multiple testing, and *p* values less than 0.05 were considered to indicate statistical significance. 

## 3. Results

We performed a comprehensive genetic assessment of DNA repair genes in 122 PrCa cases selected for very aggressive disease and 70 PrCa cases unselected for disease aggressiveness. After exclusion of 10 samples due to insufficient sequencing coverage, 122 lethal cases and 60 unselected cases remained for analysis (Figure 1)—see Table 1 for the clinical characteristics of case cohorts. 

In total, 22,850,167 variants were discovered and variant prioritization yielded 31 potentially damaging variants distributed across 17 DNA repair genes among the cases (Table 2). 

Screening of those 17 genes among the population controls revealed 157 potentially damaging variants (Supplementary Table S1) of which 137 were only discovered in the control populations, giving a total of 168 potentially damaging variants. In total, 79 of these variants were known to be pathogenic or likely pathogenic according to ClinVar, while the remaining variants were considered potentially damaging due to their truncating effects on protein domains or by having a REVEL score ≥ 0.75 and a CADD score ≥ 20. Of the 168 potentially damaging variants, 47 were classified as Tier 1 variants and 121 as Tier 2 variants. In total, 21 of the 47 Tier 1 variants were stopgain, 16 were frameshift indels, and 10 were splicing site altering variants.

In exploring the final 168 variants among the 122 lethal cases, 15 men (12.3%) carried at least one potentially damaging Tier 1 germline mutation in a DNA repair gene (one man carried two different Tier 1 mutations in the *ATM* gene), which was significantly higher than that observed in unselected cases (0%, *p* = 0.003, Table 3). 

**Figure 2:**
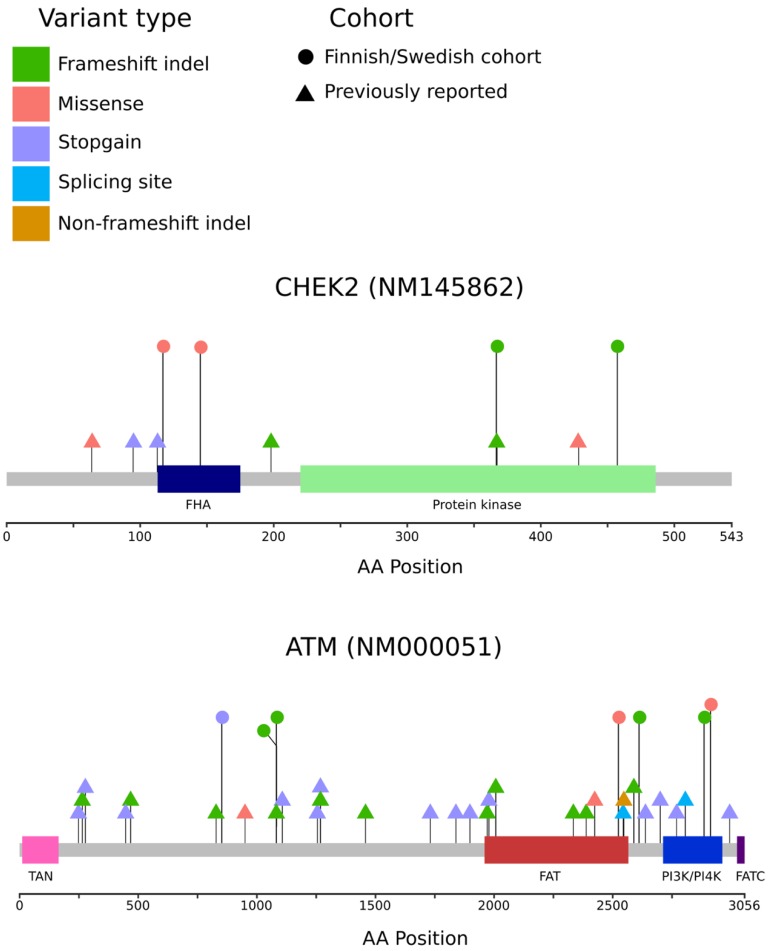
Potentially damaging variants found in the *CHEK2* and *ATM* genes. Locations of variants are shown as lollipop structures. The variants found in the Finnish/Swedish lethal or unselected cases are indicated by circles, and variants found in selected previous studies [7,11,18,28] are indicated by triangles. The variant type is indicated by the color.

No significant difference in the Tier 1 mutation carrier rate was observed between Swedish (13.3%) and Finnish (10.6%, *p* = 0.781) lethal cases. The two most frequently mutated genes were *CHEK2* (4.1%) and *ATM* (3.3%, Table 3, Figure 2). The observed carrier rate of Tier 1 mutations was significantly higher in the lethal cases compared to the prevalence in the Swedish (1.6%, *p* < 0.001) and the Finnish (5.4%, *p* = 0.040) population controls.

The observed carrier rate of potentially damaging Tier 2 germline mutations was higher in the lethal cases (13.1%) compared to that of the unselected cases (5.0%); however, the difference was not statistically significant (*p* = 0.123, Table 3). Compared to Swedish controls (6.8%, *p* = 0.011), a higher mutation rate was observed among the lethal cases; however, there was no statistically significant difference in the carrier rate of Tier 2 mutations between the lethal cases and the Finnish population controls (9.0%, *p* = 0.148). No significant difference in the Tier 2 mutation carrier rate was observed between Swedish and Finnish lethal cases (*p* = 0.102). 

No potentially damaging variants, neither Tier 1 nor Tier 2, were observed in the *BRCA2* gene in any of the PrCa cases. In the population controls, we observed a carrier rate of Tier 1 *BRCA2* mutations of 0.68% and 0.64% in Sweden and Finland, respectively.

## 4. Discussion

In this study, we characterized the germline variants occurring in the DNA repair pathway from 122 lethal and 60 unselected PrCa patients. In total, 16 potentially damaging protein truncating variants (Tier 1) were identified in 15 men (12.3%) among the lethal cases significantly exceeding the carrier rate of 0% in the unselected cases as well as the population prevalence of 1.6% and 5.4% in Swedish and Finnish population controls. In contrast, the frequency of potentially damaging nonsynonymous single nucleotide variants (Tier 2) showed similar frequencies among lethal cases, unselected cases and population controls. 

Previous studies focusing on aggressive and metastatic PrCa cases have found higher frequencies of deleterious germline variants in *BRCA2* than in any other DNA repair gene and thus considered it to be the major contributor among DNA repair genes to the aggressive phenotype [7,11,29]. However, we observed a frequency of zero pathogenic *BRCA2* variants in our lethal cases, suggesting that *BRCA2* does not play a major role in aggressive and lethal PrCa in the Swedish and Finnish populations. This agrees with earlier studies in which *BRCA1* and *BRCA2* were not found to have a significant contribution to PrCa susceptibility or aggressiveness in Finland or Sweden [30,31]. In a recent study by Mayrhofer and coworkers, sequencing of 217 metastatic PrCa cases from Sweden revealed only two pathogenic *BRCA2* mutation carriers (0.93% carrier rate, [31]). Assuming the same carrier rate among our lethal cases, we would expect to find, on average, 1.1 carriers of *BRCA2* mutations in our study, and our null finding is therefore not surprising. In general, the frequencies of established prostate cancer susceptibility variants deviate from population to population. One such case is the known cancer susceptibility variant G84E in *HOXB13,* which has been shown to have a mutation frequency approximately three-fold higher in Sweden and Finland compared to the mutation frequency in North America [32,33,34].

*ATM* and its role in pancreatic cancer was recently reviewed [35] and germline mutations in *ATM* have been associated with predisposition for several cancer forms [36] including PrCa [3]. Several studies have particularly reported potentially damaging variants in *ATM* in aggressive PrCa cases [7,9,29,31]. We also found high frequencies of potentially damaging variants in our lethal cohort (3.28% and 1.64% for Tier 1 and 2 variants, respectively), while in the unselected cases, the frequencies of these variants were found to be very low, similar to those of the population controls. These data support the evidence that deleterious variants in *ATM* are associated with the lethal phenotype of the disease. *ATM* is known to have a predominant role in the DNA damage response, but it also plays a role in maintaining the overall functionality of the cell [37]. *ATM* mutations that cause its inactivation or deficiency have shown a variety of pathological manifestations, including oxidative stress, metabolic syndrome, mitochondrial dysfunction and neurodegeneration. Recently *ATM* deficiency was shown to promote the progression of castration-resistant PrCa by enhancing the Warburg effect, suggesting that *ATM* mutation contributes through a metabolic—in addition to DNA repair—mechanism [38].

*CHEK2* variants have been associated with PrCa predisposition in several studies [9,10], and we found that this gene was the most frequently mutated Tier 1 gene in our study (4.1%). In a recent study of 217 metastatic PrCa patients from Sweden [31], *CHEK2* was also the most frequently mutated DNA repair gene (3.8%), highlighting the importance of *CHEK2* mutations for aggressive PrCa in the Nordic population. Of note, in both the present study and the study by Mayrhofer and coworkers [31], c.1100delC was the most commonly observed mutation in *CHEK2* (3.2% and 1.9%, respectively). Wu and coworkers also assessed the frequencies of potentially damaging *CHEK2* variants in lethal cases and in cases with localized low-risk PrCa from the US [39]. Overall, no association was found between *CHEK2* mutation status and lethal disease, but one variant, c.1100delC, was found to have a significantly higher frequency in the lethal cases (1.3%) compared to that of the low-risk PrCa patients (0.2%, *p* = 0.004), supporting the importance of this mutation for lethal PrCa. The c.1100delC has been shown to trigger nonsense-mediated mRNA decay, and subsequent protein analyses suggested that the truncated protein is likely highly unstable [40]. No mechanistic data are available for PrCa, but patients with *CHEK2* mutations are among those showing a high response rate to treatment with the poly-ADP ribose polymerase inhibitor Olaparib when cancers were no longer responding to standard treatments [41].

Of note, only heterozygous carriers of protein-truncating variants were observed in our study conforming to the classical two-hit model for tumor suppressor genes [42,43]. No novel candidate genes within the DNA repair pathway were found in our study. The lack of novel findings is not surprising considering the limited sample size of the study. Moreover, we applied a relatively strict approach for prioritizing variants, which may have led us to underestimate the role of some genes or even to completely miss potential candidate genes.

We pooled Finnish and Swedish lethal cases to improve the statistical power of the association analysis. No adjustment for possible confounding, for example by population stratification, PSA screening history or family history of PrCa, was performed. Population stratification is always of importance in genetic association studies. However, genotypes from genome-wide single nucleotide polymorphisms were not available for all cases and we were therefore not able to adjust for possible population stratification through principal components in the current study. PSA screening is known to decrease PrCa-specific mortality [44,45] and it is possible that screening history may have confounded our analysis. However, for this to be the case PSA screening history must be associated with carrying pathogenic mutations in DNA repair genes which we find unlikely. Finally, Pritchard and coworkers [11] reported that deleterious mutation frequencies of DNA repair genes did not differ according to whether a family history of PrCa was present among 692 men with metastatic PrCa. Therefore, we argue that confounding by family history is of limited concern in our study. 

## 5. Conclusions

In conclusion, germline variants in DNA repair genes have been shown to be associated with the aggressive form of PrCa—a finding that is supported by our study. Unlike previous studies, we did not observe high numbers of potentially damaging germline variants in *BRCA2*. Instead, mutations in *ATM* and *CHEK2* were found to be most frequent among the lethal cases, highlighting the importance of the population-specific assessment of the variants contributing to the aggressiveness of PrCa.

## Figures and Tables

**Table 1:** Clinical characteristics of patients.

	Lethal PrCa (*n* = 122)	Unselected PrCa (*n* = 60)
Age at diagnosis, median (IQR)	57.0 (55.1–58.2)	66.5 (57.8–73.8)
Diagnostic PSA level (ng/mL), median (IQR)	56.2 (17.9–247.2)	10.8 (7.0–18.8)
Clinical T-stage, *n* (%)		
TX	2 (1.8)	0 (0.0)
T1	8 (7.3)	20 (38.5)
T2	18 (16.4)	15 (28.8)
T3	61 (55.5)	15 (28.8)
T4	21 (19.1)	2 (3.8)
NA	12	8
Clinical N-stage, *n* (%)		
NX	86 (78.2)	52 (100.0)
N0	9 (8.2)	0 (0.0)
N1	15 (13.6)	0 (0.0)
NA	12	8
Clinical M-stage, *n* (%)		
MX	11 (10.0)	14 (26.9)
M0	45 (40.9)	32 (61.5)
M1	54 (49.1)	6 (11.5)
NA	12	8
Gleason score, *n* (%)		
2–6	11 (10.5)	16 (47.1)
7	36 (34.3)	7 (20.6)
8–10	58 (55.2)	11 (32.4)
NA	17	26
Death due to PrCa, n (%)	122 (100.0)	15 (25.0)
Age at death, median (IQR)	60.0 (57.9–62.9)	79.5 (69.5–84.5)

PrCa: prostate cancer; PSA: prostate-specific antigen; NA: not available.

**Table 2:** Potentially damaging mutations identified in men with lethal prostate cancer.

Gene	RSID	Type	Ref	Alt	Protein Change	ClinVar	CADD/REVEL	MAF	Tier
ATM	rs758081262	stopgain	C	T	Q852X	5	35/-	2.5 × 10^−5^	1
ATM	rs761486324	frameshift ins	-	TG	H1082fs	-	-/-	-	1
ATM	rs767099464	frameshift del	C	-	H1083fs	-	-/-	-	1
ATM	rs769142993	missense	G	C	A2524P	4	31/0.89	2.5 × 10^−5^	2
ATM	-	frameshift del	AGTAG	-	S2611fs	-	-/-	-	1
ATM	rs753961188	frameshift ins	-	T	L2885fs	5,4	-/-	4.2 × 10^−5^	1
ATM	rs376676328	missense	A	G	R2912G	3	29/0.88	3.0 × 10^−4^	2
BRCA1	rs41293459	missense	C	T	R1699Q	5,4,3	35/0.79	2.5 × 10^−5^	2
CHEK2	rs555607708	frameshift del	G	-	T367fs	5	-/-	1.8 × 10^−3^	1
CHEK2	rs137853007	missense	G	A	R145W	5,4	33/0.81	3.3 × 10^−5^	2
CHEK2	rs730881700	frameshift ins	-	T	E457fs	5,4	-/-	5.0 × 10^−5^	1
CHEK2	rs28909982	missense	T	C	R117G	5,4	27/0.93	1.0 × 10^−4^	2
ERCC3	rs753182861	frameshift del	T	-	Q586fs	-	-/-	2.0 × 10^−4^	1
ERCC3	rs145267069	missense	A	G	F297S	-	30/0.82	2.5 × 10^−5^	2
FAN1	rs778927800	missense	G	A	R749Q	-	34/0.89	8.3 × 10^−6^	2
FANCM	rs147021911	stopgain	C	T	Q1701X	4	35/0.12	1.3 × 10^−3^	1
HLTF	rs184046773	missense	C	T	G1886A	-	33/0.81	2.0 × 10^−4^	2
MRE11A	rs372000848	missense	G	A	R305W	4,3	33/0.85	5.0 × 10^−5^	2
MUTYH	rs34126013	missense	G	A	R238W	5,4	33/0.79	9.2 × 10^−5^	2
NEIL1	rs5745906	missense	G	A	G169D	-	27/0.86	1.3 × 10^−3^	2
NTHL1	rs150766139	stopgain	G	A	Q90X	5,3	35/-	1.5 × 10^−3^	1
POLG	rs761584617	missense	G	A	A1115V	-	23/0.80	2.5 × 10^−5^	2
POLG	rs113994097	missense	C	G	W748S	5,3	33/0.91	8.0 × 10^−4^	2
POLG	rs113994096	missense	G	A	P587L	5,3	28/0.80	1.7 × 10^−3^	2
POLG	rs121918052	missense	C	G	Q497H	5,3	26/0.71	2.0 × 10^−4^	2
POLL	rs139871590	missense	C	T	G356S	-	34/0.83	1.0 × 10^−3^	2
RAD18	rs138830303	stopgain	T	A	K197X	-	36/-	1.0 × 10^−4^	1
RECQL	rs149937760	missense	C	T	C414Y	-	33/0.84	2.0 × 10^−4^	2
RECQL5	rs768705080	missense	T	G	Y362S	-	32/0.76	8.2 × 10^−6^	2
TP53	rs876660754	missense	C	T	V173M	5,4	28/0.89	-	2
TP53	rs779000871	missense	G	A	T170M	3	24/0.87	8.2 × 10^−5^	2

Note: ClinVar clinical significance score defines as: 5 = pathogenic, 4 = likely pathogenic, 3 = uncertain significance. Minor allele frequency of variants derived from the Exome Aggregation Consortium. Ref: reference allele; Alt: alternative allele; ClinVar: database of reported associations of variants to clinical phenotypes; CADD: combined annotation dependent depletion; REVEL: rare exome variant ensemble learner; MAF: minor allele frequency; ins: insertion; del: deletion.

**Table 3:** Carrier rates of potentially damaging mutations, stratified by Tier 1 and Tier 2 classification, in men with lethal prostate cancer, unselected prostate cancer, and population controls.

	Lethal PrCa (*n* = 122)	Unselected PrCa (*n* = 60)	*p* Value	Finnish Controls (*n* = 3307)	*p* Value	Swedish Controls (*n* = 6192)	*p* Value
**Tier 1**							
*ERCC3, n (%)*	1 (0.82)	0	1.000	0	0.036	3 (0.05)	0.075
*RAD18, n (%)*	1 (0.82)	0	1.000	0	0.036	0	0.019
*ATM, n (%)*	4 (3.28)	0	0.304	4 (0.12)	<0.001	10 (0.16)	<0.001
*FANCM, n (%)*	2 (1.64)	0	1.000	89 (2.69)	0.772	44 (0.71)	0.223
*NTHL1, n (%)*	2 (1.64)	0	1.000	24 (0.73)	0.236	39 (0.63)	0.187
*CHEK2, n (%)*	5 (4.10)	0	0.173	60 (1.81)	0.080	5 (0.08)	<0.001
All, *n* (%)	15 (12.30)	0	0.003	177 (5.35)	0.004	101 (1.63)	<0.001
**Tier 2**							
*MUTYH, n (%)*	0	1 (1.67)	0.330	34 (1.03)	0.633	75 (1.21)	0.406
*ERCC3, n (%)*	1 (0.82)	1 (1.67)	0.552	5 (0.15)	0.195	4 (0.06)	0.093
*HLTF, n (%)*	1 (0.82)	0	1.000	20 (0.60)	0.534	9 (0.15)	0.177
*POLL, n (%)*	1 (0.82)	0	1.000	15 (0.45)	0.441	28 (0.45)	0.433
*MRE11A, n (%)*	1 (0.82)	0	1.000	0	0.036	0	0.019
*ATM, n (%)*	2 (1.64)	0	1.000	13 (0.39)	0.098	28 (0.45)	0.114
*RECQL, n (%)*	1 (0.82)	0	1.000	0	0.036	13 (0.21)	0.239
*FAN1, n (%)*	1 (0.82)	0	1.000	2 (0.06)	0.103	16 (0.26)	0.283
*NEIL1, n (%)*	1 (0.82)	0	1.000	3 (0.09)	0.135	16 (0.26)	0.283
*POLG, n (%)*	5 (4.10)	0	0.173	197 (5.96)	0.555	190 (3.07)	0.429
*TP53, n (%)*	2 (1.64)	0	1.000	3 (0.09)	0.012	7 (0.11)	0.012
*BRCA1, n (%)*	1 (0.82)	0	1.000	2 (0.06)	0.103	5 (0.08)	0.111
*RECQL5, n (%)*	1 (0.82)	0	1.000	3 (0.09)	0.135	1 (0.02)	0.038
*CHEK2, n (%)*	1 (0.82)	1 (1.67)	0.552	2 (0.06)	0.103	28 (0.45)	0.433
All, *n* (%)	16 (13.11)	3 (5.00)	0.123	299 (9.04)	0.148	420 (6.78)	0.011

PrCa: prostate cancer. P value: the frequency of potentially damaging DNA repair gene mutation carriers among the lethal PrCa patients was compared to the frequency in unselected PrCa patients and the two control populations with the use of a two-sided Fisher’s exact test.

## Supplementary Materials

The following are available online at https://www.mdpi.com/2073-4425/11/3/314/s1, Table S1: Potentially damaging variants discovered in control populations.
